# Development and Validation of an HPLC-UV Method for the Quantification of Acyclovir and Ganciclovir in the Plasma of Pediatric Immunocompromised Patients

**DOI:** 10.3390/ijms25052685

**Published:** 2024-02-26

**Authors:** Martina Franzin, Rachele Ruoso, Rossella Del Savio, Riccardo Addobbati

**Affiliations:** Institute for Maternal and Child Health, IRCCS “Burlo Garofolo”, Via dell’Istria 65/1, 34137 Trieste, Italy; martina.franzin@burlo.trieste.it (M.F.); rachele.ruoso@burlo.trieste.it (R.R.); rossella.delsavio@burlo.trieste.it (R.D.S.)

**Keywords:** therapeutic drug monitoring, pediatric, acyclovir, ganciclovir

## Abstract

Acyclovir and ganciclovir comprise the prophylaxis and treatment of herpesvirus and cytomegalovirus infections occurring in immunocompromised patients. Their therapeutic drug monitoring is fundamental because of interindividual variability leading to side effects and drug resistance and is performed through several techniques, such as liquid chromatography coupled with UV spectrophotometry (HPLC-UV) or mass spectrometry (LC-MS/MS). Therefore, we developed and validated a low-cost, non-time-consuming, and low-sample-consuming HPLC-UV method. Briefly, 100 µL of sample was used for sample preparation, mainly consisting of precipitation through organic solvent. In total, 20 µL was injected into the instrument. Chromatographic separation was obtained eluting mobile phases A (10 mM ammonium formiate 0.01% formic acid) and B (acetonitrile) on a Poroshell 120 SB-C8 2.1 × 150 mm, 2.7 µm for 12 min isocratically (97:3; A:B) at a flow rate of 0.2 mL/min. The linearity range (0.5–40 mg/L) of the method allowed us to quantify both the Cmin and Cmax of acyclovir and ganciclovir. Plasma concentrations measured on a small cohort of patients undergoing acyclovir (31) and ganciclovir (9) treatment by the proposed method and the LC-MS/MS methods, already in use, were significantly correlated. The proposed HPLC-UV method may be implemented in diagnostics as an alternative method in case of the unavailability of the LC-MS/MS system.

## 1. Introduction

Herpesvirus (HSV) and cytomegalovirus (CMV) infections occur as major complications in immunocompromised patients, leading in some cases to fatal outcomes [1,2].

To date, the gold standard prophylaxis and treatment of HSV and CMV infections consist of the antivirals acyclovir and ganciclovir. These agents are nucleoside analogues that, once converted in their corresponding triphosphates by cellular kinases, are able to incorporate themselves into viral DNA and to disrupt DNA synthesis, thus inhibiting viral replication [3].

Despite the proven efficacy of these agents, there is growing evidence of high interindividual variability in patients undergoing acyclovir and ganciclovir therapy, especially if they are pediatric patients undergoing treatment with immunosuppressive drugs [4,5]. On one hand, the dose regimen of pediatric patients is based on adult population studies and adjusted based on simple algorithms, rather than pharmacokinetic data, even if it is well known that the pharmacokinetic profile of pediatric patients differs greatly from that of adults due to anatomical and physiological factors [6]. On the other hand, therapy with immunosuppressants, such as the calcineurin inhibitors, and pre-existent pathologies could cause renal impairment, influencing drug elimination [7]. Beyond an undefined dose regimen and co-treatment with immunosuppressive drugs, genetic variants such as the *NUDT15* polymorphism could also be responsible for interindividual variability in patients undergoing acyclovir and ganciclovir treatment [8].

Acyclovir overdosing could lead to renal failure and to neuropsychiatric symptoms, such as confusion, somnolence, and hallucinations, due to the accumulation of its main metabolite 9-carboxymethoxymethylguanine; ganciclovir overdosing can cause neutropenia, thrombocytopenia, leukopenia, and elevated serum creatinine [9,10]. Notably, underdosing as an attempt to prevent the above-mentioned side effects can lead to drug resistance and therapy failure [10].

In this context, therapeutic drug monitoring is a potential solution to avoid toxicity and drug resistance and to optimize treatment in peculiar clinical scenarios. To date, target drug concentration ranges were determined for the trough concentration (Cmin) and peak concentration (Cmax) on the basis of previous studies. In particular, Maximova et al. investigated the population pharmacokinetics of intravenous acyclovir in oncologic children, defining Cmin (0.85 ± 1.3 mg/L) and Cmax (7.6 ± 5.4 mg/L) [4]. Instead, Richie and colleagues suggested 1–3 mg/L for Cmin and 3–12.5 mg/L for Cmax of ganciclovir, even if based on an adult population [5].

High-performance liquid chromatography (HPLC), coupled with several detectors, such as mass spectrometry, diode array detectors, UV spectrophotometry, and fluorescence detectors, has been applied for the quantification of acyclovir and ganciclovir in plasma and serum samples [4,10,11]. Generally, liquid chromatography coupled with mass spectrometry (LC-MS/MS) is the preferred technique to quantify these agents because of its high sensitivity, selectivity, and capability of providing quick results, even if such instrumentations are not always available and reagents employed in the analysis and sample preparation are expensive, in comparison with the conventional HPLC methods [12].

Clinical laboratories need more than one analytical method for the quantification of drugs whose therapeutic drug monitoring is necessary, so as not to discontinue the diagnostics routine and to provide rapid drug plasma concentration results to clinicians even in cases of instrumental damage. Therefore, we present a novel developed and validated method of HPLC coupled with UV spectrophotometry (HPLC-UV) for the simultaneous quantification of the antivirals acyclovir and ganciclovir, which provides comparable quantitative results to the LC-MS/MS methods used to date for diagnostics. The proposed HPLC-UV method may be implemented in clinical diagnostics routines as an alternative method in the case of the unavailability of an LC-MS/MS system, for instance as a result of instrumental damage.

## 2. Results

### 2.1. Method Development

An HPLC-UV method for the simultaneous quantification of the antivirals acyclovir and ganciclovir was compared with the LC-MS/MS methods for the quantification of each antiviral used to date for diagnostic purposes. A comparison between the features of the analytical methods is displayed in Table 1.

**Figure 1:**
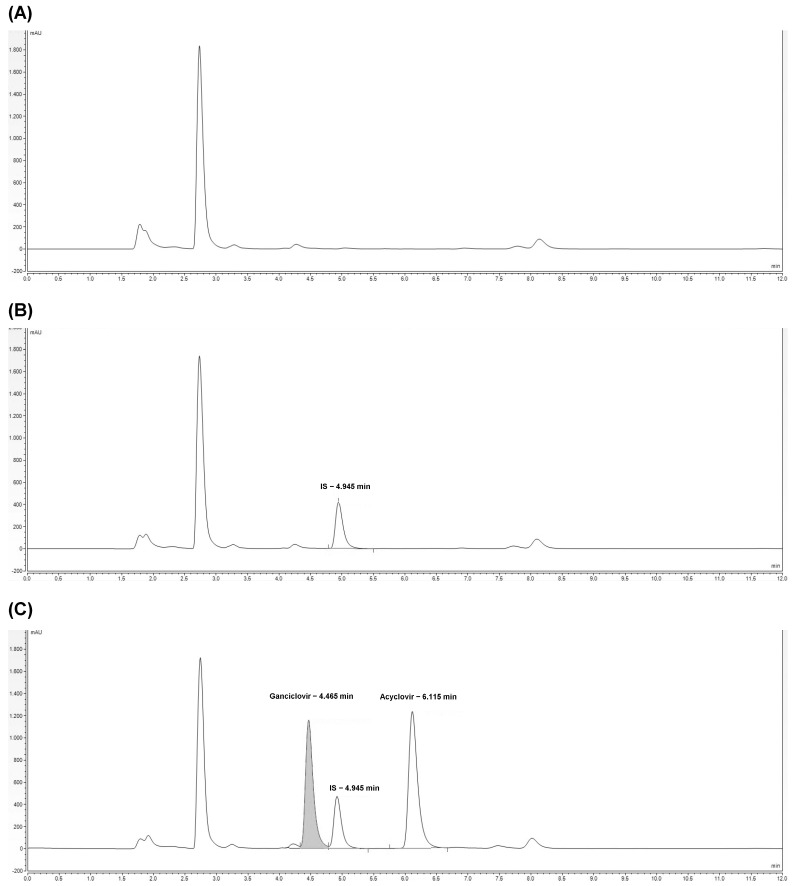
Chromatograms related to HPLC-UV analysis of plasma free drug (blank) (**A**), plasma spiked with IS (calibrator 0) (**B**), and plasma spiked with ganciclovir and acyclovir (quality control at a concentration of 20 µg/mL) (**C**). The retention times of IS, ganciclovir, and acyclovir are reported.

As shown in Figure 1, the proposed HPLC-UV method has demonstrated no interference of compounds belonging to the biological matrix at the same retention time of the analytes and internal standard (IS) at the wavelength used for the analysis In detail, the retention times of acyclovir, ganciclovir, and IS are 6.1, 4.5, and 4.9 min, respectively.

Chromatograms related to the LC-MS/MS analysis showing the background noise, acyclovir, and ganciclovir are reported in Figure S1.

Sample purification using Phree Phospholipid Removal Products, capable of removing both proteins and phospholipids, was also tested without any benefit; on the contrary, the signals related to the analytes and the IS were reduced.

### 2.2. Method Validation

#### 2.2.1. Linearity

The linear regression model confirmed the linearity of the HPLC-UV method in the range of concentrations tested (0.5–40 µg/mL). The coefficient of determination (R2) for all the three calibration curves was greater than 0.99 for both acyclovir and ganciclovir quantification. Furthermore, the within-run and between-run percentages of accuracy of the calibrators (CAL) were estimated: the calculated concentration resulted in accurate values (100 ± 15% of the nominal concentration) (Table 2).

The within-run and between-run percentages of accuracy of the CAL analyzed by LC-MS/MS are shown in Table S1.

#### 2.2.2. Sensitivity

Sensitivity was evaluated by analyzing dilutions of the calibrator with the lowest concentration and determining the lower limit of quantification (LLOQ) and detection (LOD) values, intended as the lowest concentration quantifiable and detectable. In particular, diluting CAL1 resulted in a loss of accuracy in quantification and a loss of signal for both acyclovir and ganciclovir; therefore, LLOQ and LOD correspond to CAL1 (0.5 µg/mL). Instead, LLOQ and LOD values for the quantification of both acyclovir and ganciclovir referred to the LC-MS/MS analyses resulted in values of 0.5 and 0.05 µg/mL, respectively.

#### 2.2.3. Accuracy and Precision

The within-run and between-run accuracy and precision of three levels of quality controls (QC) were also estimated to assess an accurate and reproducible quantification of the antivirals. As shown in Table 3, the percentage of accuracy was 100 ± 15% of the nominal concentration, which in our opinion does not exceed the threshold value established by the ICH guidelines [13]. Also, CV% was far lower than 15%.

The within-run and between-run percentages of accuracy and CV% of QCs analyzed by LC-MS/MS are shown in Table S2.

#### 2.2.4. Matrix Effect and Recovery

In order to allow the reliability of results and efficiency of the analytical procedure, the matrix effect (ME), recovery of the extraction procedure (RE) and the overall process efficiency (PE) were evaluated (Table 4). Notably, there was no signal suppression due to the biological matrix in all of the three levels of concentration tested. Furthermore, the recovery of the analytes after sample preparation and analysis was >90%. Therefore, the process efficiency, the overall parameter obtained from the previous ones, resulted in optimal values.

Indications for ME, RE, and PE related to the LC-MS/MS analyses were reported in Table S3.

#### 2.2.5. Specificity

The interference of another immunosuppressive drug, mycophenolate, which is administered sometimes in pediatric oncologic patients and whose therapeutic drug monitoring is usually performed in plasma, was also tested by injecting a pure standard added with an IS. As shown in Figure S2, the retention time of mycophenolate is 1.8 min, different from the analytes and IS.

### 2.3. Method Applicability

Plasma concentrations of acyclovir and ganciclovir were obtained respectively pre and post-dose from 31 and 9 samples of pediatric patients undergoing intravenous therapy. Both the HPLC-UV and LC-MS/MS methods were used for quantification. In detail, the median and the interquartile range (IQR) related to acyclovir measurements was 1.42 mg/L (IQR: 0.81–5.50) for HPLC-UV analysis and 1.16 mg/L (IQR: 0.53–4.90) for LC-MS/MS analysis. Instead, the median and the IQR related to ganciclovir measurements was 4.26 mg/L (IQR: 0.35–6.46) for HPLC-UV analysis and 3.37 mg/L (IQR: 0.39–8.10) for LC-MS/MS analysis.

**Figure 2:**
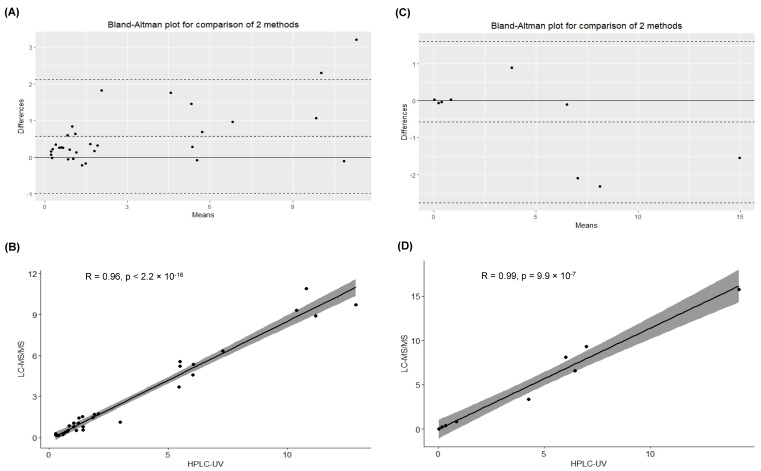
(**A**) Bland–Altman plot evaluating the differences between the data obtained for the quantification of acyclovir by the analytical methods. (**B**) Spearman correlation test to evaluate the association between the calculated concentrations (mg/L) of acyclovir in the samples (n = 31) quantified by HPLC-UV and LC-MS/MS (*p* < 2.2 × 10^−16^). (**C**) Bland–Altman plot evaluating the differences between the data obtained for the quantification of ganciclovir by the analytical methods. (**D**) Pearson correlation test to evaluate the association between the calculated concentrations (mg/L) of ganciclovir in the samples (n = 9) quantified by HPLC-UV and LC-MS/MS (*p* < 9.9 × 10^−7^).

Bland–Altman plots describe the agreement between the quantitative measurements between the methods. In particular, Figure 2A,C display a scatter diagram of the differences plotted against the averages of the two measurements. Horizontal lines represent the mean difference and the limits of agreement (±1.96 SD of differences). Notably, the plasma concentration of drugs obtained from the different analyses showed no significant biases.

Furthermore, Spearman and Pearson correlation tests described a significant positive correlation for acyclovir and ganciclovir quantification, respectively (Spearman correlation test for acyclovir quantification: *p* < 2.2 × 10^−16^; Pearson correlation test for ganciclovir quantification: *p* < 9.9 × 10^−7^), as shown in Figure 2B,D.

## 3. Discussion

Since there is a need to have analytical methods which are not cost-, time-, and sample-consuming, with instrumentation easily available in every laboratory, and to overcome delays in providing results to clinicians in cases of instrumental damage, we have developed and validated an HPLC-UV method for the simultaneous quantification of the antivirals acyclovir and ganciclovir. Indeed, comparing the features of the HPLC-UV method with the LC-MS/MS methods already used for diagnostic purposes, we noticed several advantages consisting in the cost of reagents, the availability of the instrumentation, the volume of sample needed, the introduction of bromouracil as an IS, and the isocratic elution. In particular, the cost of reagents was greatly influenced by the choice of the stable isotope IS in LC-MS/MS, which have almost identical chemical properties [14]. 

Interestingly, since bromouracil was used in our work as an IS for the quantification of both acyclovir and ganciclovir in the proposed HPLC-UV method, the simultaneous quantification of these analytes was allowed by HPLC-UV. Instead, currently, the quantification of acyclovir and ganciclovir is performed by two LC-MS/MS methods, having the same instrumental conditions, but using each IS for the normalization of the response of the other. The introduction of bromouracil as an IS, and therefore the simultaneous quantification of both antivirals, could allow the use of the same calibrators and quality controls, allowing us to reduce the costs and the timing of the analysis. Techniques different from HPLC-UV are employed to obtain this purpose [15,16,17,18]; instead, to the authors’ knowledge, only a few papers have described HPLC-UV methods able to quantify both of these antivirals, as the chromatographic separation of these antivirals is hard to obtain due to their similar chemical structure [19,20,21]. Among the limitations of our study, there is the impossibility of quantifying 9-carboxymethoxymethylguanine, the main metabolite of acyclovir responsible for side effects encountered after antiviral treatment, as found in another study conducted previously [18].

Contrary to the manuscript from Shibata and colleagues, the proposed method was also validated in order to be implemented in clinical diagnostic routine [21]. Validation parameters were in line with those recommended by the ICH guidelines and by Matuszewski, thus suggesting the analytical method showed an appropriate sensitivity, linearity, accuracy, precision, and efficiency of the process [13,22,23]. Nonetheless, the analytical method could be improved to be more sensitive: other previous studies have described HPLC-UV methods with a lower LOD [19,20]. Anyway, our method is able to quantify both acyclovir and ganciclovir up to 0.5 mg/L, well below the Cmin reported in the scientific literature [4,5]. Notably, the linearity range assessed by our method was larger and covered the concentrations expected by Cmin and Cmax for acyclovir and ganciclovir, differently from the other studies [19,20].

Since the antivirals tested were administered in immunocompromised patients undergoing treatment with immunosuppressive drugs, we considered the interference of one of these agents in the optimization of the method. Interestingly, several immunosuppressive drugs, such as cyclosporine A, tacrolimus, sirolimus, and everolimus, were measured in whole blood and were not present in plasma after deproteinization, thus they were not potentially interfering with the analysis [24]. On the contrary, therapeutic drug monitoring of mycophenolate is generally performed in plasma and, for this reason, we decided to test the drug interference of this agent [25]. Notably, mycophenolate elutes at a different retention time from the analytes of interest, indicating that the proposed HPLC-UV method could also be performed in cases of co-treatment with this immunosuppressant.

As previously conducted [15], a small cohort of patients undergoing intravenous acyclovir (31) or ganciclovir (9) treatment was also investigated to assess the applicability of the novel HPLC-UV method. Notably, the quantitative results obtained with the proposed HPLC-UV method were significantly correlated and comparable with the ones obtained from the LC-MS/MS analyses actually used for diagnostic purposes.

Our study presents some limitations. In particular, based on the ICH Q2(R2) guideline on the validation of analytical procedures, the evaluation of accuracy and precision was performed by testing three levels of concentrations across the reportable range in triplicate, differently from what was suggested by the ICH guideline M10 on bioanalytical method validation and study sample analysis, Step 5 (four concentration levels and five replicates per level) [13,23].

In conclusion, beyond developing and validating a novel HPLC-UV method for the simultaneous quantification of acyclovir and ganciclovir, we tested the agreement between the novel analytical method and the LC-MS/MS methods actually used in our laboratory. Based on the results obtained from validation but particularly from quantitative data on real samples, the novel HPLC-UV may be implemented as an alternative method in the case of the unavailability of the LC-MS/MS system, for instance following instrumental damage.

Interesting new research insights would be gained from analyzing the plasma concentrations of the antivirals acyclovir and ganciclovir administered intravenously in cohorts of pediatric patients, taking into consideration their age, genotype, and dose.

## 4. Materials and Methods

### 4.1. Chemicals and Reagents

All chemicals and reagents used were of analytical grade. Pure water was obtained from a Milli-Q system (Millipore, Darmstadt, Germany). Acyclovir, ganciclovir, bromouracil, methanol, acetonitrile, trifluoracetic acid, ammonium formiate, and formic acid were purchased by Sigma-Aldrich (Milan, Italy). Phree Phospholipid Removal Products were purchased from Phenomenex (Bologna, Italy).

### 4.2. Biological Samples

Peripheral blood samples (3 mL) were collected in EDTA tubes from pediatric patients (0–18 years) undergoing intravenous acyclovir (n = 31) and ganciclovir (n = 9) therapeutic drug monitoring and were left over from routine clinical analysis. The use of leftover samples for the validation of analytical methods for the improvement of therapeutic drug monitoring was approved by IRCCS Burlo Garofolo (RC 56/22). Peripheral blood samples were collected after at least 5 days of antiviral treatment before the morning dose (trough concentration) or 30 min after the end of the infusion (peak concentration). Plasma was obtained by centrifugation at 3000× *g* for 5 min and stored at −80 °C until the following analysis for up to 1 month.

### 4.3. Stocking and Working Solutions

Free-drug plasma samples were obtained from healthy subjects and stored at −80 °C. Stock solutions, comprising acyclovir and ganciclovir dissolved in MilliQ water (1 mg/mL), were prepared and aliquoted at −80 °C for up to 12 months, a period in which they were certified to be stable. Working solutions A (500 µg/mL acyclovir) and B (500 µg/mL ganciclovir) were prepared by diluting the stock solutions 1:2 in MilliQ water on the day of the analysis. Moreover, CAL and QC were prepared by spiking the working solutions in free-drug plasma to achieve the final concentrations. In detail, a calibration curve comprising 6 calibration points (0.5, 1, 5, 10, 25, and 40 µg/mL) and 3 levels of QC (2.5, 12.5, 20 µg/mL) was constructed using different working solutions. A solution of bromouracil in methanol (100 µg/mL) was used as an IS for the quantification by HPLC-UV. Instead, regarding the LC-MS/MS analysis for the quantification of acyclovir, the working solution B was used as an IS; instead, regarding the LC-MS/MS analysis for the quantification of ganciclovir, the working solution A was used as an IS.

### 4.4. HPLC-UV Analysis

Regarding the sample preparation for HPLC-UV analysis, 25 µL of IS and 175 µL of methanol were added to 100 µL of CAL/QC/sample to allow deproteinization. After centrifugation for 5 min at 12,100× *g*, 250 µL of supernatant was collected, dried under a gentle stream of nitrogen, and subsequently resuspended in 50 µL of MilliQ water. Sample purification using Phree Phospholipid Removal Products was also tested. In total, 20 µL was injected into the instrument for the analysis.

The HPLC-UV method for the quantification of the antivirals acyclovir and ganciclovir was developed on a UPLC UltiMate™ 3000 coupled with the Dionex™ UltiMate™ 3000 VWD-3000 (Thermo Fischer Scientific, Milan, Italy). Separation was obtained by eluting mobile phases A (10 mM ammonium formiate 0.01% formic acid) and B (acetonitrile) on a Poroshell 120 SB-C8 2.1 × 150 mm, 2.7 µm (Agilent Technologies, Milan, Italy) for 12 min isocratically (97:3; A:B) at a flow rate of 0.2 mL/min. The column temperature was set at 25 °C. The wavelength set up for the analysis was 250 nm. Data acquisition and processing was performed using the Chromeleon™ Chromatography Data System version 7.0 (Thermo Fischer Scientific, Milan, Italy).

### 4.5. LC-MS/MS Analyses

Regarding the sample preparation for LC-MS/MS analyses, 10 µL of IS and 50 µL of a solution of 20% trifluoracetic acid were added to 150 µL of CAL/QC/sample. After centrifugation for 5 min at 121,00× *g*, 10 µL of supernatant was diluted with 90 µL of 0.1% formic acid in acetonitrile, and subsequently 5 µL was injected into the instrument.

The LC-MS/MS method for the quantification of the antivirals acyclovir and ganciclovir, used for the data comparation with the HPLC-UV method, was developed on a Shimadzu LC-40D XR coupled with a triple quad Sciex 3200. Separation for each molecule was obtained likewise, eluting mobile phases A (2 mM ammonium formiate 0.2% formic acid) and B (2 mM ammonium formiate 0.2% formic acid in acetonitrile) on an Allure PFPP 2.1 × 50 mm, 5 µm (Restek srl, Milan, Italy) at a flow rate of 0.4 mL/min with a gradient. In particular, the gradient used was 0–0.5 min, 5% B; 1 min, 10% B; 1.6 min, 98% B; 1.9–2.2 min, 2% B; 4.0 min, stop. The column temperature was set at 25 °C. The electrospray ionization was set to positive mode. The mass spectrometer operated in multiple reaction monitoring (MRM) mode controlled by Analyst operating software version 1.4 (Sciex, Milan, Italy). The ion spray voltage was set to 5000 V and the source temperature to 500 °C. Nitrogen was used as the collision gas. The nebulizer (GS1), curtain, and turbo gas (GS2) were set to 50, 25, and 60 psi, respectively. MRM parameters of analytes were optimized as described in Table 5. Dwell times were set to 75 ms for each transition.

### 4.6. Analytical Validation

Validation of the analytical method was performed using the International Council for Harmonisation of Technical Requirements for Pharmaceuticals for Human Use (ICH) guidelines [13,23]. Briefly, the selectivity and specificity of the analytical method were assessed testing blank samples; linearity was determined through the construction of a 6-point calibration curve within a run in 3 different runs; sensitivity was assessed through the dilution of the calibrator at the lowest concentration and the evaluation of LLOQ and LOD. The within-run and between-run accuracy, measured as the percentage of accuracy, and precision, measured as CV%, were estimated by the use of 3 levels of QC. Interferences in the analytical method due to co-treatment with immunosuppressants (mycophenolate) were also investigated. Instead, the ME, RE, and process efficiency were determined using a pool of plasma samples obtained from 6 individuals and performing experiments in triplicate, as previously described by Matuszewski [22].

### 4.7. Statistical Analysis

Concentration was calculated by normalizing the response ratio of analytes to that of IS; calibration curves were fit by linear regression with 1/ꭕ 2) weighting. In order to compare the concentrations of samples quantified by the analytical methods, Pearson and Spearman correlation tests, depending on data distribution, were performed using R software version 3.0.

## Figures and Tables

**Table 1:** Comparison of the features of the HPLC-UV method developed with the LC-MS/MS methods, already in use for diagnostic purposes.

Features	LC-MS/MS Methods (Already in Use)	HPLC-UV Method (Developed)
Reagents and instrumentation costs	High	Low
Availability of instrumentation used	Less frequently available	More frequently available
Volume of plasma sample	150 µL	100 µL
Internal standard used	Acyclovir for ganciclovir quantification; ganciclovir for acyclovir quantification	Bromouracil
Time for sample preparation	15 min	20 min
Injected volume	5 µL	20 µL
Time for sample analysis	4 min	12 min
Type of elution of mobile phases	Gradient	Isocratic

**Table 2:** Within-run (1 injection) and between-un accuracy (%) of CAL referring to acyclovir and ganciclovir quantification by HPLC-UV. RSD%: relative standard deviation %.

Acyclovir									
Standard	Nominal Concentration (µg/mL)	1		2		3		Between-Run Accuracy (%)	Between-Run Means (µg/mL) (RSD%)
		Calculated Concentration (µg/mL)	Within-Run Accuracy (%)	Calculated Concentration (µg/mL)	Within-Run Accuracy (%)	Calculated Concentration (µg/mL)	Within-Run Accuracy (%)		
CAL1	0.5	0.503	99.40	0.496	100.80	0.499	100.20	100.13	0.499 (0.70%)
CAL2	1	0.990	101.00	1.010	99.00	1.010	99.00	99.67	1.003 (1.15%)
CAL3	5	4.843	103.14	5.161	96.78	5.132	97.36	99.09	5.045 (3.48%)
CAL4	10	10.793	92.07	9.841	101.59	9.781	102.19	98.62	10.138 (5.60%)
CAL5	25	24.844	100.62	25.082	99.67	24.919	100.32	100.21	24.948 (0.49%)
CAL6	40	39.083	102.29	39.273	101.82	39.014	102.47	102.19	39.123 (0.34%)
Ganciclovir									
Standard	Nominal Concentration (µg/mL)	1		2		3		Between-Run Accuracy (%)	Between-Run Means (µg/mL) (RSD%)
		Calculated Concentration (µg/mL)	Within-Run Accuracy (%)	Calculated Concentration (µg/mL)	Within-Run Accuracy (%)	Calculated Concentration (µg/mL)	Within-Run Accuracy (%)		
CAL1	0.5	0.494	101.20	0.501	99.80	0.466	106.80	102.60	0.487 (3.80%)
CAL2	1	1.014	98.60	0.990	101.00	0.943	105.70	101.77	0.982 (3.68%)
CAL3	5	4.974	100.52	5.171	96.58	5.03	99.40	98.83	5.058 (2.00%)
CAL4	10	10.757	92.43	9.942	100.58	9.693	103.07	98.69	10.131 (5.49%)
CAL5	25	24.491	102.04	25.109	99.56	24.517	101.93	101.18	24.706 (1.41%)
CAL6	40	38.502	103.75	39.136	102.16	38.227	104.43	103.45	38.622 (1.21%)

**Table 3:** Within-run (3 injections) and between-run accuracy (%) and precision, expressed as the coefficient of variation (CV%) of the QC referring to acyclovir and ganciclovir quantification by HPLC-UV. SD: standard deviation.

Acyclovir													
Standard	Nominal Concentration (µg/mL)	1			2			3			Between-Run Accuracy (%)	Between Run Precision (CV%)	Between-Run Means (µg/mL) (SD)
		Within-Run Accuracy (%)	Within-Run Precision (CV%)	Within-Run Means (µg/mL) (SD)	Within-Run Accuracy (%)	Within-Run Precision (CV%)	Within-Run Means (µg/mL) (SD)	Within-Run Accuracy (%)	Within-Run Precision (CV%)	Within-Run Means (µg/mL) (SD)			
QCI	2.5	100.75	5.79	2.55 (0.15)	99.20	5.84	2.63 (0.15)	99.42	5.81	2.62 (0.15)	99.79	5.81	2.60 (0.04)
QCII	12.5	96.38	2.34	13.20 (0.31)	96.04	0.02	13.49 (0.01)	94.67	2.34	13.64 (0.32)	95.70	1.57	13.44 (0.22)
QCIII	20	99.86	0.37	20.11 (0.07)	97.84	0.37	20.92 (0.08)	98.17	0.37	20.78 (0.08)	98.62	0.37	20.60 (0.43)
Ganciclovir													
Standard	Nominal Concentration (µg/mL)	1			2			3			Between-Run Accuracy (%)	Between-Run Precision (CV%)	Between-Run Means (µg/mL) (SD)
		Within-Run Accuracy (%)	Within-Run Precision (CV%)	Within-Run Means (µg/mL) (SD)	Within-Run Accuracy (%)	Within-Run Precision (CV%)	Within-Run Means (µg/mL) (SD)	Within-Run Accuracy (%)	Within-Run Precision (CV%)	Within-Run Means (µg/mL) (SD)			
QCI	2.5	105.66	5.68	2.32 (0.13)	99.45	5.36	2.61 (0.14)	101.20	5.40	2.53 (0.14)	102.10	5.48	2.48 (0.15)
QCII	12.5	98.41	2.11	12.71 (0.27)	94.81	2.09	13.63 (0.28)	96.12	2.09	13.29 (0.28)	96.44	2.10	13.21 (0.46)
QCIII	20	101.65	0.48	19.41 (0.09)	98.34	0.48	20.73 (0.10)	99.58	0.48	20.24 (0.10)	99.86	0.48	20.12 (0.67)

**Table 4:** Matrix effect (ME), recovery (RE), and process efficiency (PE) calculated on QC referring to the HPLC-UV method.

Standard	Acyclovir			Ganciclovir		
	ME (%)	RE (%)	PE (%)	ME (%)	RE (%)	PE (%)
QCI	95.69	94.89	90.80	99.53	91.53	91.10
QCII	101.57	95.99	97.49	101.64	94.55	96.10
QCIII	93.36	98.73	92.17	91.90	97.28	89.40

**Table 5:** Optimized compound parameters for MRM detection of quantifier (_1) and qualifier ions (_2/_3) including mass selected in the first quadrupole (Q1 mass) and in the third quadrupole (Q3 mass), declustering potential (DP), entrance potential (EP), collision energy (CE), and cell exit potential (CXP).

ID	Q1 Mass (Da)	Q3 Mass (Da)	DP (V)	EP (V)	CE (V)	CXP (V)
Acyclovir_1	226.100	152.100	60	10	20	5
Acyclovir_2	226.100	135.100	60	10	35	5
Acyclovir_3	226.100	110.100	60	10	35	5
Ganciclovir_1	256.100	152.100	60	10	20	5
Ganciclovir_2	256.100	135.100	60	10	35	5
Ganciclovir_3	256.100	110.100	60	10	35	5

## Data Availability

Data are available upon reasonable request to the corresponding author.

## Supplementary Materials

The following supporting information can be downloaded at: https://www.mdpi.com/article/10.3390/ijms25052685/s1.
