# Supplementary material for: Development and Validation of an HPLC-UV Method for the Quantification of Acyclovir and Ganciclovir in the Plasma of Pediatric Immunocompromised Patients
Source: Int J Mol Sci. 2024 Feb 26;25(5):2685. doi: 10.3390/ijms25052685 (PMC10932297; doi:10.3390/ijms25052685)
Supplement: Supplementary file 1 [file ijms-25-02685-s001.zip › Tables S1_S3 revised.pdf]

Table S1. Within-run (1 injection) and between-run accuracy (%) of CAL referred to acyclovir and ganciclovir quantification by LC-MS/MS. RSD% relative standard deviation %.

Acyclovir									
Standard	Nominal concentration (μg/mL)	1		2		3		Between-run accuracy (%)	Between-run means (RSD %)
		Calculated concentration (μg/mL)	Within-run accuracy (%)	Calculated Concentration (μg/mL)	Within-run accuracy (%)	Calculated Concentration (μg/mL)	Within-run accuracy (%)		
CAL1	0.5	0.549	109.71	0.528	105.61	0.542	108.42	107.91	0.540 (1.98%)
CAL2	1	1.026	102.58	0.922	92.19	0.968	96.78	97.18	0.972 (5.36%)
CAL3	5	4.657	93.14	5.147	102.93	4.760	95.20	97.09	4.855 (5.32%)
CAL4	10	9.036	90.36	10.064	100.64	9.781	97.81	96.27	9.627 (5.52%)
CAL5	25	25.753	103.01	24.350	97.40	25.443	101.77	100.73	25.182 (2.93%)
CAL6	40	40.480	101.20	40.488	101.22	40.008	100.02	100.81	40.325 (0.68%)
**Ganciclovir**									
Standard	Nominal concentration (μg/mL)	1		2		3		Between-run accuracy (%)	Between-run means (RSD %)
		Calculated concentration (μg/mL)	Within-run accuracy (%)	Calculated Concentration (μg/mL)	Within-run accuracy (%)	Calculated Concentration (μg/mL)	Within-run accuracy (%)		
CAL1	0.5	0.487	97.47	0.515	102.96	0.492	98.39	99.61	0.498 (3.00%)

CAL2	1	1.048	104.75	0.941	94.08	1.025	102.50	100.44	1.005 (5.61%)
CAL3	5	5.129	102.57	4.943	98.85	5.159	103.18	101.53	5.077 (2.30%)
CAL4	10	9.922	99.22	10.127	101.27	10.282	102.82	101.10	10.110 (1.79%)
CAL5	25	24.590	98.36	25.500	102.00	24.580	98.32	99.56	24.890 (2.12%)
CAL6	40	39.052	97.63	40.336	100.84	37.916	94.79	97.75	39.101 (3.10%)

Table S2. Within-run (3 injections) and between-run accuracy (%) and precision (CV%) of QC referred to acyclovir and ganciclovir quantification by LC-MS/MS.

Acyclovir													
		1			2			3					
Standard	Nominal concentration (μg/mL)	Within-run accuracy (%)	Within-run precision (CV%)	Within-run means (μg/mL) (SD)	Within-run accuracy (%)	Within-run precision (CV%)	Within-run means (μg/mL) (SD)	Within-run accuracy (%)	Within-run precision (CV%)	Within-run means (μg/mL) (SD)	Between-run accuracy (%)	Between-run precision (CV%)	Between-run means (μg/mL) (SD)
QCI	2.5	94.48	2.66	2.36 (0.06)	99.70	1.64	2.49 (0.04)	94.70	2.70	2.37 (0.06)	96.29	2.33	2.37 (0.01)
QCII	12.5	95.20	2.45	11.9 (0.29)	98.52	3.37	12.32 (0.42)	100.38	1.61	12.55 (0.20)	98.03	2.48	12.23 (0.46)
QCIII	20	98.76	2.13	19.75 (0.42)	101.76	2.04	20.35 (0.42)	99.59	0.45	19.92 (0.09)	100.04	1.54	19.84 (0.12)
Ganciclovir													
		1			2			3					
Standard	Nominal concentration (μg/mL)	Within-run accuracy (%)	Within-run precision (CV%)	Within-run means (μg/mL) (SD)	Within-run accuracy (%)	Within-run precision (CV%)	Within-run means (μg/mL) (SD)	Within-run accuracy (%)	Within-run precision (CV%)	Within-run means (μg/mL) (SD)	Between-run accuracy (%)	Between-run precision (CV%)	Between-run means (μg/mL) (SD)
QCI	2.5	104.29	0.77	2.61 (0.02)	100.37	4.48	2.51 (0.11)	101.63	4.32	2.54 (0.11)	102.10	3.19	2.55 (0.05)
QCII	12.5	102.60	3.39	12.83 (0.44)	100.49	1.64	12.56 (0.21)	98.84	1.63	12.35 (0.20)	100.64	2.22	12.58 (0.24)
QCIII	20	99.96	1.01	19.99 (1.01)	100.02	1.55	20.00 (0.31)	98.10	1.55	19.62 (0.30)	99.36	1.37	19.87 ()

Table S3. Matrix effect (ME), recovery (RE) and process efficiency (PE) calculated on QC referred to the LC-MS/MS methods.

**Standard**	**Acyclovir**			**Ganciclovir**		
	**ME (%)**	**RE (%)**	**PE (%)**	**ME (%)**	**RE (%)**	**PE (%)**
QCI	93.96	89.55	84.14	101.77	97.26	98.98
QCII	102.26	90.62	92.67	101.20	99.06	100.24
QCIII	105.55	90.66	95.70	95.28	99.08	94.41
