# Supplementary figures and images for: Development and Validation of an HPLC-UV Method for the Quantification of Acyclovir and Ganciclovir in the Plasma of Pediatric Immunocompromised Patients
Source: Int J Mol Sci. 2024 Feb 26;25(5):2685. doi: 10.3390/ijms25052685 (PMC10932297; doi:10.3390/ijms25052685)

Ganciclovir

A)

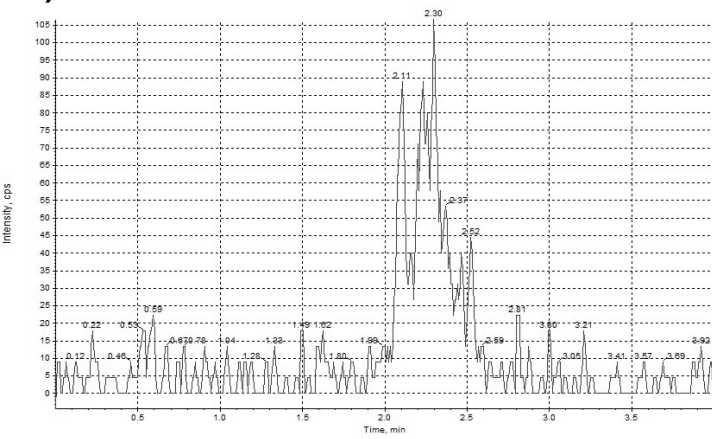

Acyclovir

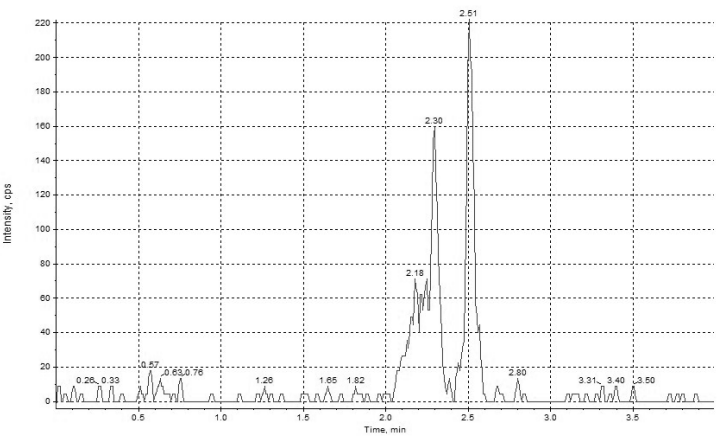

B)

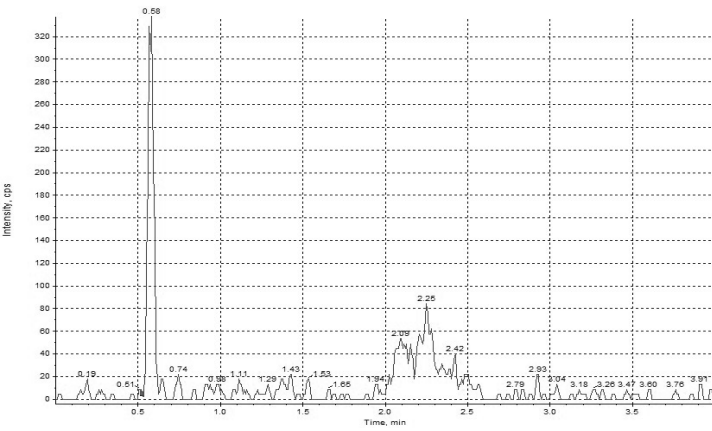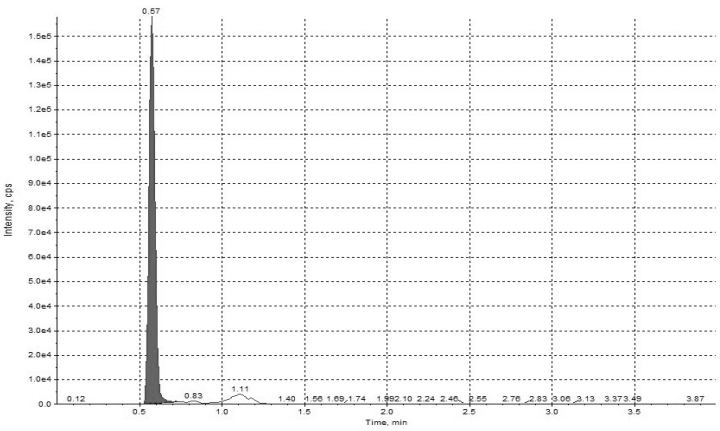

C)

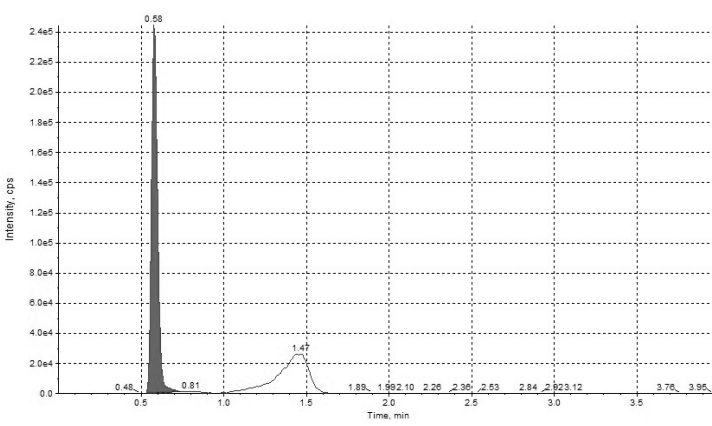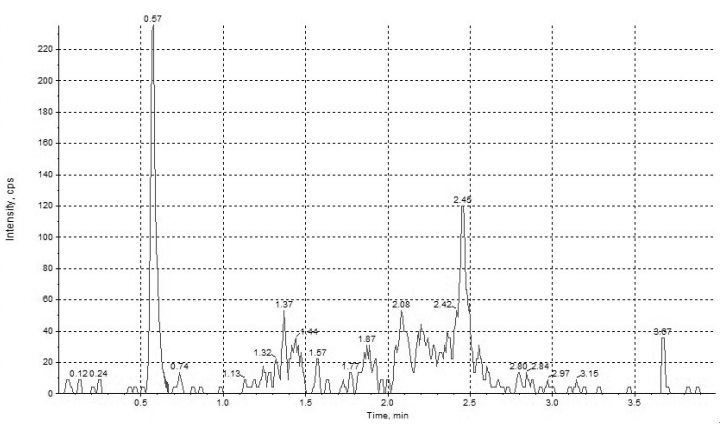

D)

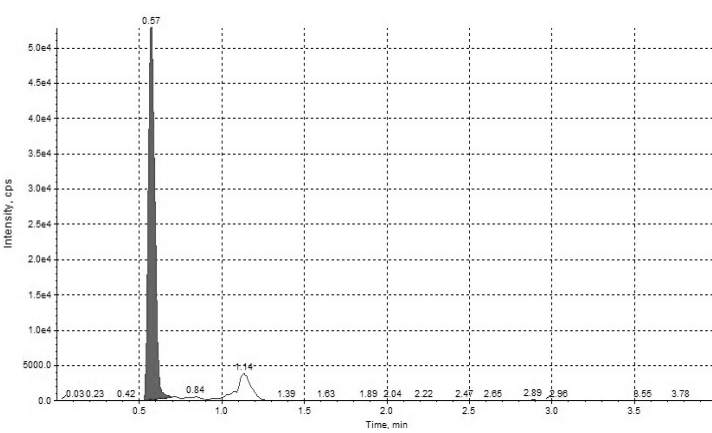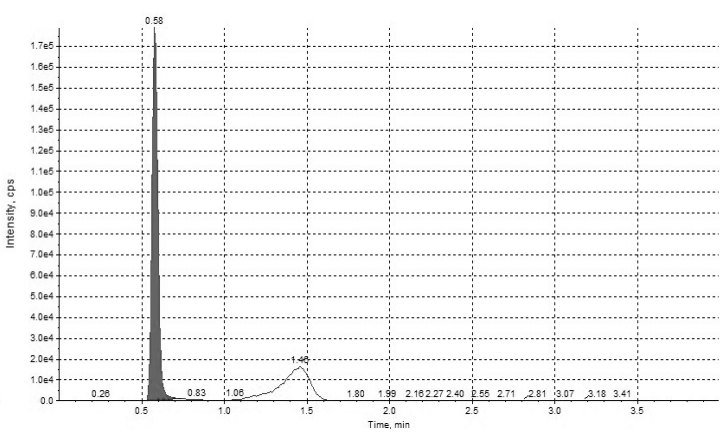

Supplement: Supplementary file 1 [file ijms-25-02685-s001.zip › Figure S1.pdf]

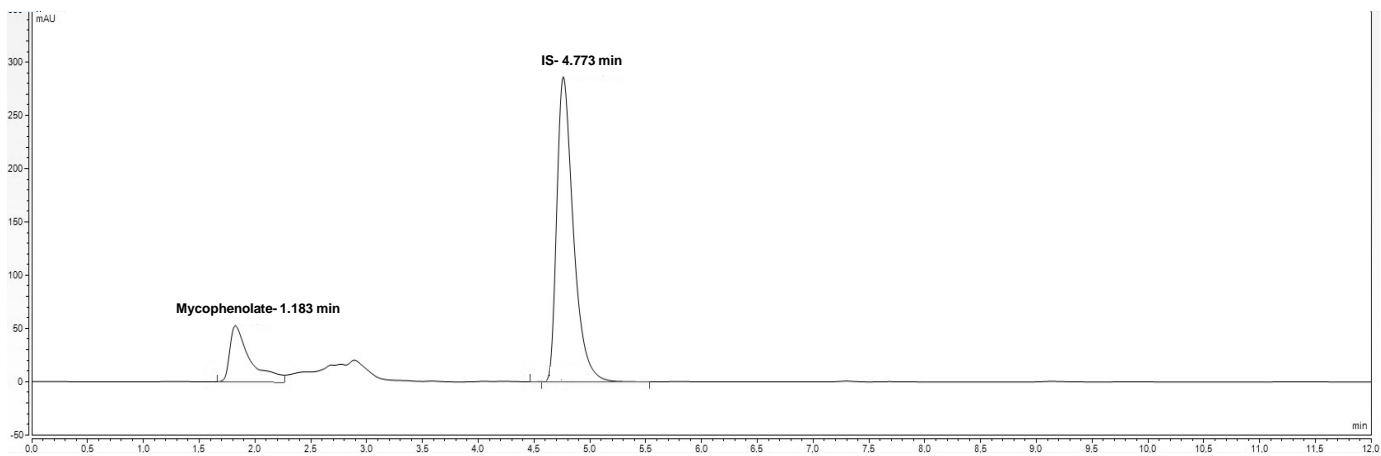

Supplement: Supplementary file 1 [file ijms-25-02685-s001.zip › Figure S2.pdf]
